# Impact of Maternal Obesity on Delivery Outcomes Following Labor Induction: A Single‐Center Retrospective Cohort Study

**DOI:** 10.1111/jog.70282

**Published:** 2026-04-15

**Authors:** Shina Sakaguchi, Eishin Nakamura, Shigetaka Matsunaga, Akihiko Kikuchi, Yasushi Takai

**Affiliations:** ^1^ Center for Maternal, Fetal and Neonatal Medicine, Saitama Medical Center Saitama Medical University Saitama Japan; ^2^ Department of Obstetrics and Gynecology, Saitama Medical Center Saitama Medical University Saitama Japan

**Keywords:** body mass index, labor induction, obesity, oxytocin, pregnancy

## Abstract

**Aim:**

This study evaluated the association between body mass index (BMI) at the onset of labor induction and delivery outcomes, including instrumental delivery, emergency cesarean section, labor duration, and cumulative oxytocin dose among singleton term pregnancies.

**Methods:**

We conducted a retrospective cohort study of singleton term pregnancies undergoing labor induction at a tertiary perinatal center. We analyzed BMI at induction onset as a continuous variable and applied multivariable regression models to evaluate instrumental delivery, emergency cesarean section, labor duration, and cumulative oxytocin dose. We also performed sensitivity analyses using pre‐pregnancy BMI, models additionally adjusting for gestational weight gain and pregnancy complications, and restricted‐indication analyses.

**Results:**

Delivery BMI was not significantly associated with instrumental delivery (adjusted OR 0.96 per 1 kg/m^2^ increase; 95% CI 0.92–1.00), emergency cesarean section (adjusted OR 1.03; 95% CI 0.99–1.07), or labor duration. In contrast, cumulative oxytocin dose increased with higher delivery BMI (*β* = 0.04 per 1 kg/m^2^ increase; 95% CI 0.01–0.07), corresponding to approximately 20% greater oxytocin requirement per 5 kg/m^2^ increment in this cohort. This pattern persisted across sensitivity analyses.

**Conclusions:**

Higher maternal BMI at induction onset was associated with greater oxytocin requirements, while no clear associations were observed with mode of delivery or labor duration.

## 1 Introduction

Maternal obesity, defined as a body mass index (BMI) of ≥ 30.0 kg/m^2^ before or in early pregnancy, has emerged as a major public health concern [1]. The global prevalence of maternal obesity increased markedly from approximately 5% before 1990 to about 21% in 2024, and projections estimate that it will reach nearly 23% by 2030 [2]. As the number of women with obesity continues to rise, obstetric care increasingly encounters pregnancies complicated by maternal obesity.

Maternal obesity is associated with a wide range of adverse perinatal conditions, including gestational diabetes mellitus (GDM) and hypertensive disorders of pregnancy (HDP) [3]. These complications often necessitate management in tertiary care centers and contribute to increased obstetric complexity. Consequently, understanding how maternal obesity affects intrapartum management and delivery outcomes remains an important clinical issue.

Previous studies have reported an association between maternal obesity and higher cesarean delivery rates, particularly among nulliparous women undergoing labor induction [4, 5, 6]. However, evidence regarding the effect of obesity on delivery outcomes after induction—such as instrumental delivery, emergency cesarean section, and labor progression—remains inconsistent across studies [4, 5, 7, 8]. These discrepancies likely reflect differences in study populations, obesity severity, and definitions of maternal obesity.

Most prior research has defined maternal obesity using pre‐pregnancy or early‐pregnancy BMI [3, 4, 5, 6]. In contrast, maternal body size at the time of induction reflects the clinical condition at the start of intrapartum management. Few studies have specifically examined BMI at the initiation of labor induction in relation to delivery outcomes [9, 10]. Therefore, we evaluated the association between BMI at the onset of induction and delivery outcomes in this cohort.

Accordingly, we aimed to evaluate, among singleton term pregnancies undergoing labor induction, the association between BMI at the onset of induction—analyzed as a continuous variable—and instrumental delivery, emergency cesarean section, labor duration, and cumulative oxytocin dose.

## 2 Methods

### 2.1 Study Design and Setting

We conducted this retrospective cohort study at Saitama Medical Center, Saitama Medical University, a tertiary perinatal center, between April 1, 2019, and March 31, 2023. During the study period, the institution provided comprehensive perinatal care, including 24‐h intrapartum management. We included all eligible singleton term pregnancies that underwent labor induction during this period.

### 2.2 Participants

We included all singleton term pregnancies in which clinicians initiated labor induction at our institution. We excluded women with multiple pregnancies and those who underwent elective cesarean section without an attempt at labor induction.

### 2.3 Exposure

We defined delivery BMI as the primary exposure and analyzed it as a continuous variable. Delivery BMI was calculated as maternal body weight at delivery (kg) divided by height squared (m^2^), reflecting maternal body size at the initiation of labor induction and intrapartum management. In supplementary analyses, pre‐pregnancy BMI was calculated as pre‐pregnancy weight divided by height squared (m^2^) and analyzed as a continuous variable.

### 2.4 Outcomes

We defined instrumental delivery and emergency cesarean section as the primary outcomes. At our institution, clinicians perform instrumental delivery exclusively using forceps. We defined labor duration and cumulative oxytocin dose as secondary outcomes. We measured labor duration as the interval from the initiation of induction—such as premature rupture of membranes, cervical ripening, cervical dilation, onset of uterine contractions, or administration of uterotonic agents—to delivery. We calculated cumulative oxytocin dose from the documented infusion rate and duration in the electronic medical records.

### 2.5 Confounders

We selected infertility treatment, parity, and intrapartum analgesia or anesthesia as potential confounders based on clinical relevance. Clinicians provided analgesia or anesthesia at maternal request or when medically indicated, including for cardiac disease, cerebrovascular disease, or HDP. Analgesia or anesthesia included epidural, spinal, or intravenous methods. GDM was diagnosed according to the International Association of Diabetes and Pregnancy Study Groups criteria using a 75‐g oral glucose tolerance test [11]. HDP was defined according to the criteria of the American College of Obstetricians and Gynecologists (Practice Bulletin No. 222) [12].

### 2.6 Data Sources and Measurements

We extracted clinical data from electronic medical records. Pre‐pregnancy weight was obtained from self‐reported values documented in the standardized intake questionnaire at the first prenatal visit. For cervical ripening and dilation, clinicians used magnesium sulfate–containing polyvinyl alcohol preparations, double‐balloon catheters, or intravaginal dinoprostone inserts. Clinicians initiated oxytocin infusion at 2 mU/min and increased the dose by 2 mU/min at intervals of 30 min or longer, up to a maximum of 20 mU/min. They reduced or discontinued the infusion when uterine tachysystole or non‐reassuring fetal heart rate patterns occurred. We used a standardized oxytocin augmentation protocol at our institution across all BMI levels. Decisions regarding emergency cesarean delivery or instrumental delivery were based on established clinical indications and were not altered solely according to maternal BMI.

### 2.7 Statistical Analysis

We categorized indications for labor induction as post‐term pregnancy or premature rupture of membranes, uterine inertia (primary or secondary), pregnancy complications, or other indications. We compared categorical variables using the *χ* ^2^ test and continuous variables using the Wilcoxon rank‐sum test. For the primary outcomes—instrumental delivery and emergency cesarean section—we performed multivariable logistic regression analyses and calculated odds ratios (ORs) with 95% confidence intervals (CIs). For the secondary outcomes—labor duration and cumulative oxytocin dose—we conducted multivariable linear regression analyses and estimated regression coefficients (β) with 95% CIs. Baseline continuous variables were presented as mean ± standard deviation to facilitate comparison between groups, whereas variables with skewed distributions or outcome‐related variables were presented as median (interquartile range), as appropriate.

We performed sensitivity analyses as follows:

1. analyses using pre‐pregnancy BMI;
2. analyses adjusting simultaneously for pre‐pregnancy BMI and gestational weight gain;
3. analyses adjusting for birth weight, including an exploratory interaction term between delivery BMI and birth weight in the multivariable logistic regression model for instrumental delivery, as instrumental delivery may be more directly influenced by fetal size than other outcomes;
4. analyses additionally adjusting for pregnancy complications and maternal factors potentially affecting mode of delivery (GDM, HDP, fetal growth restriction [FGR], contracted pelvis, maternal comorbidities requiring medical management, and fetal anomalies);
5. analyses restricted to cases induced for post‐term pregnancy, premature rupture of membranes, or primary or secondary uterine inertia to reduce heterogeneity in induction indications and potential confounding by indication by excluding medically indicated early inductions, and to explore the robustness of the findings within a more clinically homogeneous subgroup;
6. analyses restricted to women who achieved vaginal delivery to assess whether the association between delivery BMI and cumulative oxytocin dose was influenced by delivery course;
7. stratified analyses by pre‐pregnancy BMI categories (< 25 and ≥ 25 kg/m^2^) to evaluate whether the association between delivery BMI and outcomes differed by maternal baseline body size, using the same multivariable models as in the primary analysis.

We conducted all statistical tests as two‐sided analyses and considered *p* values < 0.05 statistically significant. We performed all statistical analyses using RStudio (version 2024.04.0) with R (version 4.4.0).

## Ethics Statement

The Ethics Committee of Saitama Medical Center, Saitama Medical University approved this study (approval number: 2024–102). Because of the retrospective design, the committee waived the requirement for written informed consent, and the institution provided an opt‐out procedure on its website.

## 3 Results

### 3.1 Study Population

**FIGURE 1:**
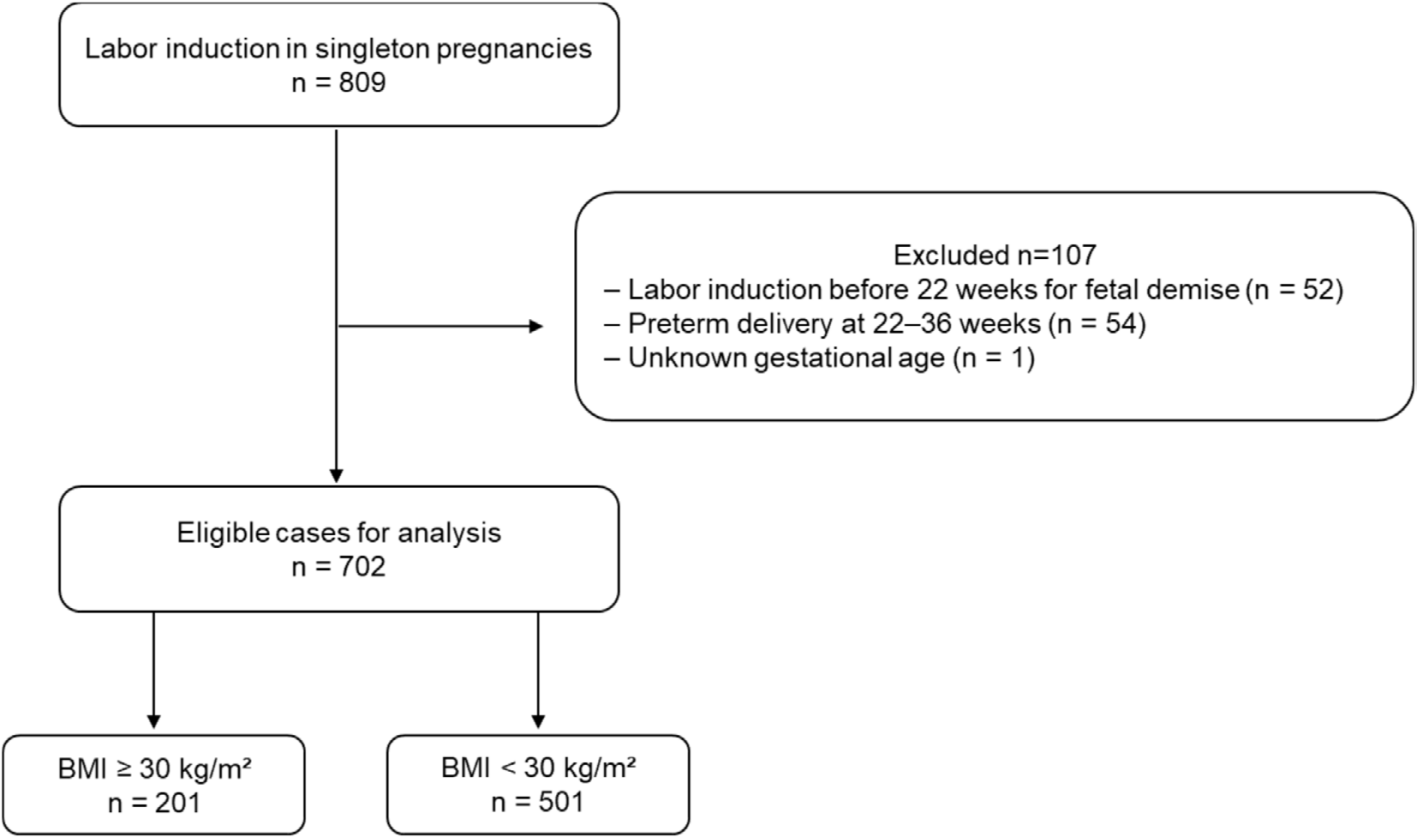
Flow diagram of the patient study cohort. During the study period, 3837 deliveries were performed at our institution. Among singleton term pregnancies, labor induction was performed in 809 women. After excluding ineligible cases, including multiple pregnancies and elective cesarean sections, a total of 702 women were included in the final analysis.

During the study period, our institution recorded 3837 deliveries. Among these, clinicians performed labor induction in 809 singleton term pregnancies. After excluding cases with incomplete clinical information, we included 702 women in the final analysis (Figure 1).

### 3.2 Baseline Characteristics

We compared baseline characteristics between women with delivery BMI < 30 kg/m^2^ and those with delivery BMI ≥ 30 kg/m^2^. Women in the delivery BMI ≥ 30 kg/m^2^ group had a significantly higher prevalence of GDM and HDP. In addition, the distribution of indications for labor induction differed significantly between the two groups (Table 1).

**TABLE 1:** Baseline characteristics according to delivery BMI.

Characteristic	BMI < 30 kg/m^2^ (*N* = 501)	BMI ≥ 30 kg/m^2^ (*N* = 201)	*p*
A. Maternal characteristics			
Delivery BMI (kg/m^2^), median (IQR)	25 (23–27)	33 (31–36)	—
Age (years)	33.9 ± 5.5	33.3 ± 5.5	0.267
Multiparity, *n* (%)	144 (28.7%)	61 (30.3%)	0.741
Epidural analgesia, *n* (%)	261 (52.1%)	84 (41.8%)	0.017
Infertility treatment, *n* (%)	187 (37.3%)	50 (24.9%)	< 0.001
B. Pregnancy complications			
GDM, *n* (%)	42 (8.4%)	43 (21.4%)	< 0.001
HDP, *n* (%)	26 (5.2%)	24 (11.9%)	< 0.001
FGR, *n* (%)	10 (2.0%)	4 (2.0%)	1.000
C. Indications for labor induction			< 0.001
Post‐term/PROM, *n* (%)	180 (35.9%)	103 (51.2%)	—
Labor dysfunction, *n* (%)	253 (50.5%)	71 (35.3%)	—
Maternal/fetal complications, *n* (%)	57 (11.4%)	21 (10.4%)	—
Others, *n* (%)	11 (2.2%)	6 (3.0%)	—
D. Delivery outcomes			
Forceps delivery, *n* (%)	114 (22.8%)	25 (12.4%)	0.003
Emergency cesarean section, *n* (%)	78 (15.6%)	36 (17.9%)	0.517

*Note:* Overall forceps delivery was 139/702 (19.8%), and overall emergency cesarean section was 114/702 (16.2%). Delivery BMI is presented as median (IQR); ranges were 17–29 kg/m^2^ for the BMI < 30 group and 30–50 kg/m^2^ for the BMI ≥ 30 group. Values are presented as mean ± SD or number (%), as appropriate. *p* values represent unadjusted comparisons between BMI categories and were calculated using the Wilcoxon rank‐sum test for continuous variables and the *χ* ^2^ test or Fisher's exact test for categorical variables. Indications for labor induction were categorized as follows: post‐term/PROM included post‐term pregnancy and premature rupture of membranes; labor dysfunction included primary and secondary uterine inertia; maternal/fetal complications included HDP, FGR, other maternal complications (including contracted pelvis or suspected cephalopelvic disproportion), suspected macrosomia, fetal anomalies, and fetal indications (e.g., abnormal fetal surveillance); others included social or non‐medical reasons for induction. For indications for labor induction, the *p* value represents comparison of the overall distribution between groups.

Abbreviations: BMI, body mass index; FGR, fetal growth restriction; GDM, gestational diabetes mellitus; HDP, hypertensive disorders of pregnancy; PROM, premature rupture of membranes.

### 3.3 Primary Outcomes

In multivariable logistic regression analyses treating delivery BMI as a continuous variable, delivery BMI was not significantly associated with instrumental delivery (adjusted odds ratio [OR] 0.96, 95% confidence interval [CI] 0.92–1.00; *p* = 0.061). Similarly, delivery BMI was not significantly associated with emergency cesarean section (adjusted OR 1.03, 95% CI 0.99–1.07; *p* = 0.185) (Table 2).

**TABLE 2:** Association between delivery BMI and labor outcomes.

Outcome	Adjusted estimate (95% CI)	*p*
Primary outcomes		
Forceps delivery	OR 0.96 (0.92–1.00)	0.061
Emergency cesarean section	OR 1.03 (0.99–1.07)	0.185
Secondary outcomes		
Log labor duration	*β* 0.01 (−0.00–0.03)	0.185
Log cumulative oxytocin dose	*β* 0.04 (0.01–0.07)	0.004

*Note:* Delivery BMI was analyzed as a continuous variable. Adjusted estimates were obtained from multivariable logistic regression models for binary outcomes and linear regression models for continuous outcomes. Models were adjusted for maternal age, parity, epidural analgesia, and infertility treatment. For binary outcomes, results are presented as odds ratios (ORs) with 95% confidence intervals (CIs). For continuous outcomes, results are presented as regression coefficients (*β*) with 95% CIs after logarithmic transformation of the outcome variables.

Abbreviation: BMI, body mass index.

### 3.4 Secondary Outcomes

**FIGURE 2:**
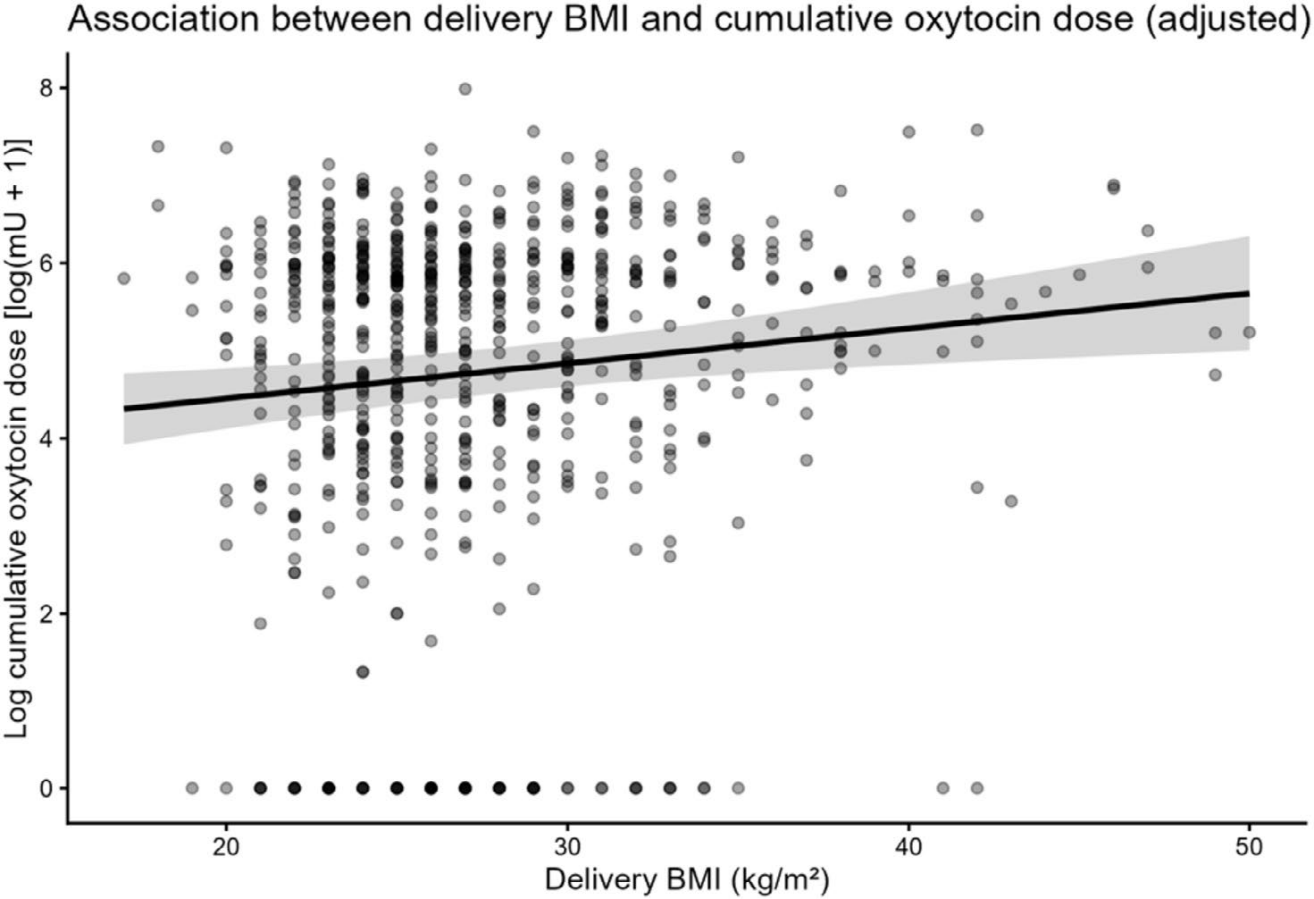
Association between delivery body mass index and cumulative oxytocin dose. Each dot represents an individual case. The solid line represents the adjusted regression line derived from a multivariable linear regression model, and the shaded area indicates the 95% confidence interval. The model was adjusted for maternal age, parity, epidural analgesia, and infertility treatment.

Delivery BMI did not show a significant association with labor duration. In contrast, higher delivery BMI was significantly associated with a higher cumulative oxytocin dose during labor (regression coefficient *β* = 0.04, 95% CI 0.01–0.07; *p* = 0.004) (Table 2). This association remained significant after adjustment for maternal age at delivery, parity, epidural analgesia, and infertility treatment. As shown in Figure 2, delivery BMI was positively associated with cumulative oxytocin dose.

### 3.5 Sensitivity Analyses

We summarized maternal weight–related characteristics according to delivery BMI in Table S1. Women with delivery BMI ≥ 30 kg/m^2^ had significantly higher pre‐pregnancy BMI but significantly lower gestational weight gain than women with delivery BMI < 30 kg/m^2^ (both *p* < 0.001).

In sensitivity analyses using pre‐pregnancy BMI alone (Table S2), pre‐pregnancy BMI showed a statistically significant inverse association with instrumental delivery. However, when pre‐pregnancy BMI and gestational weight gain were simultaneously included in the model (Table S3), this association was no longer statistically significant. No statistically significant associations were observed for emergency cesarean section or labor duration in either model. In contrast, the positive association between BMI and cumulative oxytocin dose remained statistically significant across these models.

When we additionally adjusted for birth weight, we did not observe evidence of interaction between delivery BMI and birth weight with respect to instrumental delivery (Table S4). Additional adjustment for GDM, HDP, FGR, maternal comorbidities, and fetal anomalies did not materially alter the main findings: the positive association between delivery BMI and cumulative oxytocin dose persisted, whereas no clear associations emerged with instrumental delivery or emergency cesarean section (Table S5). We then restricted the analysis to inductions for post‐term pregnancy, PROM, and primary or secondary uterine inertia (*n* = 607) (Table S6). In this restricted cohort, delivery BMI was significantly associated with instrumental delivery (OR 0.95, 95% CI 0.90–0.99), emergency cesarean section (OR 1.05, 95% CI 1.00–1.10), and labor duration. In contrast, the association between delivery BMI and cumulative oxytocin dose remained significant and comparable in magnitude to that observed in the primary analysis. We further restricted the analysis to women who achieved vaginal delivery (*n* = 588) (Table S7). In this subgroup, delivery BMI remained significantly associated with cumulative oxytocin dose (*β* = 0.036 per 1 kg/m^2^ increase, 95% CI 0.007–0.066; *p* = 0.014). Finally, we performed stratified analyses according to pre‐pregnancy BMI categories (< 25 and ≥ 25 kg/m^2^) (Table S8). In women with pre‐pregnancy BMI < 25 kg/m^2^, delivery BMI was significantly associated with emergency cesarean section, whereas no such association was observed in women with pre‐pregnancy BMI ≥ 25 kg/m^2^. Conversely, the association between delivery BMI and cumulative oxytocin dose was observed only among women with pre‐pregnancy BMI ≥ 25 kg/m^2^.

## 4 Discussion

In this study of singleton term pregnancies undergoing labor induction, we evaluated delivery BMI as a continuous variable and examined its association with delivery outcomes. In the primary analyses, delivery BMI was not significantly associated with instrumental delivery, emergency cesarean section, or labor duration. Pre‐pregnancy BMI showed a statistically significant inverse association with instrumental delivery, whereas results for other outcomes were similar. In contrast, higher delivery BMI was consistently associated with greater cumulative oxytocin dose across all sensitivity analyses. In stratified analyses according to pre‐pregnancy BMI categories, the associations between delivery BMI and outcomes differed across strata. Specifically, delivery BMI was associated with emergency cesarean section among women with pre‐pregnancy BMI < 25 kg/m^2^, whereas its association with cumulative oxytocin dose was observed only among those with pre‐pregnancy BMI ≥ 25 kg/m^2^ (Table S8). These findings suggest that pre‐pregnancy BMI and delivery BMI may capture different aspects of maternal physiology, and that their associations with labor outcomes may not be uniform across BMI strata. However, these stratified analyses were exploratory and should be interpreted with caution.

Previous studies have reported mixed findings regarding the association between maternal obesity and delivery outcomes after labor induction [4, 5, 6, 7, 8]. Several studies found no clear association between obesity and instrumental delivery [4, 7], whereas others reported higher cesarean delivery rates among women with obesity, particularly in Western populations [5, 6]. In the primary analysis using delivery BMI as a continuous variable, instrumental delivery was not statistically significant (OR 0.96 per 1 kg/m^2^ increase; 95% CI 0.92–1.00; *p* = 0.061). In the analysis using pre‐pregnancy BMI, instrumental delivery reached statistical significance (OR 0.96; 95% CI 0.92–1.00; *p* = 0.046). In both models, the upper bound of the confidence interval was 1.00. Because statistical significance differed between delivery BMI and pre‐pregnancy BMI, we did not consider instrumental delivery to be consistently associated with BMI in this cohort.

Several factors may explain why higher BMI was not associated with an increased rate of emergency cesarean section in our cohort. First, many previous studies included a substantial proportion of women with severe obesity (e.g., BMI ≥ 35–40) [5, 6], whereas extremely high BMI values were less common in our cohort. Differences in BMI distribution may reduce the likelihood of detecting differences in emergency cesarean section rates. Second, decisions to proceed to emergency cesarean section after labor induction depend on institutional resources and intrapartum management strategies. Our institution primarily manages high‐risk pregnancies and provides advanced intrapartum care with continuous 24‐h monitoring, allowing clinicians to tolerate longer labor durations before proceeding to cesarean delivery. This institutional context may have contributed to the comparable delivery outcomes observed across BMI levels. In addition, active nutritional counseling and weight management during pregnancy may have influenced gestational weight gain patterns. Third, BMI correlates with multiple clinical factors related to delivery outcomes. After accounting for these factors in sensitivity analyses, BMI alone did not independently determine emergency cesarean section risk.

We also did not observe a significant association between delivery BMI and labor duration, which aligns with previous reports [8]. In contrast, higher delivery BMI was consistently associated with increased oxytocin requirements, consistent with previous reports [9, 10]. In addition to confirming previous reports, this study evaluated both pre‐pregnancy BMI and BMI at the time of induction within the same cohort, allowing assessment of their respective associations with oxytocin requirement and delivery outcomes under a standardized induction protocol. This association persisted after adjustment for pre‐pregnancy BMI, gestational weight gain, birth weight, and pregnancy complications. Higher delivery BMI was associated with approximately 4% greater cumulative oxytocin dose per 1 kg/m^2^, corresponding to roughly a 20% increase for a 5 kg/m^2^ difference and about a 50% increase for a 10 kg/m^2^ difference. Based on the median cumulative oxytocin dose of 205 mU in our cohort, this translates to an estimated increase of approximately 45 mU for a 5 kg/m^2^ difference and approximately 100 mU for a 10 kg/m^2^ difference.

We therefore conducted sensitivity analyses to assess how changes in analytical conditions might influence the observed associations. In the analysis additionally adjusted for pregnancy‐related complications (Table S5), the association between delivery BMI and cumulative oxytocin dose remained significant, indicating that this relationship was not explained solely by these complications. In the restricted analysis excluding inductions for maternal or fetal indications and limited to post‐term pregnancy, PROM, and uterine inertia (Table S6), delivery BMI was significantly associated with instrumental delivery (OR 0.95, 95% CI 0.90–0.99), emergency cesarean section (OR 1.05, 95% CI 1.00–1.10), and labor duration. These findings differed from the primary analysis, in which these associations were not statistically significant. Because this restricted analysis was designed to reduce heterogeneity in induction indications and to examine the robustness within a more clinically homogeneous subgroup, the differences from the primary analysis likely reflect variation in case mix rather than a consistent overall effect of BMI. Moreover, as multiple outcomes were examined across several analytical specifications, some statistically significant findings may be sensitive to model specification. Because the primary analysis included the full cohort and was prespecified as the main analysis, we considered it the principal estimate of association. In contrast, the association between delivery BMI and cumulative oxytocin dose was consistent across analyses, suggesting that this represents the most robust finding of the study. Because cumulative oxytocin dose may depend on delivery course and could be truncated in cases converted to emergency cesarean section, we additionally restricted the analysis to women who achieved vaginal delivery (*n* = 588). In this subgroup, delivery BMI remained significantly associated with cumulative oxytocin dose (*β* = 0.036 per 1 kg/m^2^ increase, 95% CI 0.007–0.066; *p* = 0.014) (Table S7). Overall, these findings indicate that associations with mode of delivery and labor duration varied according to analytical specification, whereas the association with cumulative oxytocin dose showed a consistent pattern across analyses.

Several biological mechanisms may account for the increased oxytocin requirements observed in women with higher delivery BMI. Increased volume of distribution in women with obesity may delay attainment of effective circulating oxytocin concentrations. In addition, experimental studies indicate that adipose tissue– and placenta‐derived leptin can attenuate myometrial contractility, potentially reducing oxytocin responsiveness [13, 14]. However, the clinical relevance of these mechanisms in humans remains uncertain and warrants further investigation.

In countries such as the United States and the United Kingdom, where maternal obesity is more prevalent, clinical guidelines emphasize comprehensive management of obesity in pregnancy, including weight management and preparation for delivery [15, 16]. However, these settings often include a higher proportion of severe obesity and report greater differences in delivery outcomes [4, 5, 6]. In our cohort managed under current protocols at a Japanese tertiary care center, delivery outcomes were broadly comparable between women with and without obesity, despite higher oxytocin requirements with increasing delivery BMI. These findings indicate that, under a standardized induction protocol, higher maternal BMI is associated with increased oxytocin requirements without clear differences in delivery outcomes. Nevertheless, given the consistently higher oxytocin requirements observed, further investigation in prospective studies is warranted.

This study has several strengths. It is among the few studies to evaluate BMI at the onset of labor induction, a clinically relevant time point for intrapartum management [9, 10]. Moreover, multiple sensitivity analyses incorporating alternative BMI measures, gestational weight gain, pregnancy complications, and birth weight supported the robustness of the main findings. In addition, both pre‐pregnancy BMI and BMI at induction were evaluated within the same cohort under a standardized induction protocol.

Several limitations warrant consideration. As a retrospective single‐center study, residual confounding and institutional practice patterns may have influenced the results. Although clinicians followed standardized management protocols, individual clinical judgment guided oxytocin titration and decisions regarding operative delivery. In addition, delivery BMI may not be directly comparable to pre‐pregnancy BMI used in many prior studies [5, 6]. Future multicenter prospective studies should validate these findings and help establish optimized induction strategies for women with obesity.

In conclusion, in the primary analysis, mode of delivery was largely comparable across BMI levels, whereas higher delivery BMI was associated with increased cumulative oxytocin requirements during labor induction under a standardized management protocol. The clinical implications of these findings for individualized intrapartum management require further investigation.

## Author Contributions

**Shina Sakaguchi:** data curation, formal analysis, writing – original draft. **Eishin Nakamura:** writing – review and editing, formal analysis, supervision, validation. **Shigetaka Matsunaga:** conceptualization, methodology, supervision. **Akihiko Kikuchi:** conceptualization, methodology, supervision. **Yasushi Takai:** conceptualization, methodology, supervision.

## Ethics Statement

The Ethics Committee of Saitama Medical Center, Saitama Medical University approved this study (approval number: 2024‐102).

## Consent

The authors have nothing to report.

## Conflicts of Interest

The authors declare no conflicts of interest or financial disclosures relevant to the content of this manuscript. Yasushi Takai is an Editorial Board member of the Journal of Obstetrics and Gynecology Research and a co‐author of this article. To minimize he was excluded from all editorial decision‐making related to the acceptance of this manuscript.

## Supporting information


**Table S1:** Maternal weight‐related characteristics according to delivery body mass index.
**Table S2:** Association between pre‐pregnancy body mass index and labor outcomes.
**Table S3:** Association between pre‐pregnancy body mass index and labor outcomes adjusted for gestational weight gain.
**Table S4:** Multivariable logistic regression including an interaction term between delivery BMI and birth weight for instrumental delivery.
**Table S5:** Sensitivity analyses adjusting for pregnancy complications.
**Table S6:** Sensitivity analyses restricted to inductions for post‐term pregnancy, premature rupture of membranes, and uterine inertia (*n* = 607).
**Table S7:** Association between delivery BMI and cumulative oxytocin dose restricted to vaginal deliveries (*n* = 588).
**Table S8:** Stratified analyses according to pre‐pregnancy BMI categories.

## Data Availability

The data underlying this study consist of individuallevel clinical information derived from electronic medical records at a single tertiary perinatal center. Due to ethical restrictions approved by the institutional review board and the risk of patient reidentification, these data cannot be made publicly available in an open repository. However, deidentified aggregate data and analysis code used in this study may be shared upon reasonable request to the corresponding author, subject to institutional approval.
