# Supplementary material for: Impact of Maternal Obesity on Delivery Outcomes Following Labor Induction: A Single‐Center Retrospective Cohort Study
Source: J Obstet Gynaecol Res. 2026 Apr 15;52(4):e70282. doi: 10.1111/jog.70282 (PMC13082913; doi:10.1111/jog.70282)
Supplement: Supplementary file 1 — Table S1: Maternal weight‐related characteristics according to delivery body mass index. Table S2: Association between pre‐pregnancy body mass index and labor outcomes. Table S3: Association between pre‐pregnancy body mass index and labor outcomes adjusted for gestational weight gain. Table S4: Multivariable logistic regression including an interaction term between delivery BMI and birth weight for instrumental delivery. Table S5: Sensitivity analyses adjusting for pregnancy complications. Table S6: Sensitivity analyses restricted to inductions for post‐term pregnancy, premature rupture of membranes, and uterine inertia (n = 607). Table S7: Association between delivery BMI and cumulative oxytocin dose restricted to vaginal deliveries (n = 588). Table S8: Stratified analyses according to pre‐pregnancy BMI categories. [file JOG-52-0-s001.docx]

**Supplementary Table S1. Maternal weight–related characteristics according to delivery body mass index**

| **Characteristic** | **BMI <30 kg/m²** | **BMI ≥30 kg/m²** | **p value** |
| --- | --- | --- | --- |
| Pre-pregnancy BMI, *kg/m²* | 20.8 (19.2–22.4) | 29.2 (26.3–32.8) | < 0.001 |
| Gestational weight gain, *%* | 19 (13.5–24) | 12.3 (4.7–21) | < 0.001 |

Values are presented as median (interquartile range).

P values were calculated using the Wilcoxon rank-sum test.

P values less than 0.001 are presented as p < 0.001.

BMI, body mass index.

**Supplementary Table S2. Association between pre-pregnancy BMI and labor outcomes**

| **Outcome** | **Adjusted estimate (95% CI)** | **p value** |
| --- | --- | --- |
| **Primary outcomes** |  |  |
| Instrumental delivery | OR 0.96 (0.92–1.00) | 0.046 |
| Emergency cesarean section | OR 1.01 (0.98–1.05) | 0.475 |
| **Secondary outcomes** |  |  |
| Log labor duration | β 0.01 (−0.00–0.02) | 0.196 |
| Log cumulative oxytocin dose | β 0.03 (0.01–0.06) | 0.013 |

Pre-pregnancy BMI was analyzed as a continuous variable.

Adjusted estimates were obtained from multivariable logistic regression models for binary outcomes and linear regression models for continuous outcomes.

Models were adjusted for maternal age, parity, epidural analgesia, and infertility treatment.

For binary outcomes, results are presented as odds ratios (ORs) with 95% confidence intervals (CIs).

For continuous outcomes, results are presented as regression coefficients (β) with 95% CIs after logarithmic transformation of the outcome variables.

BMI, body mass index.

**Supplementary Table S3. Association between pre-pregnancy BMI and labor outcomes adjusted for gestational weight gain**

| **Outcome** | **Adjusted estimate (95% CI)** | **p value** |
| --- | --- | --- |
| **Primary outcomes** |  |  |
| Instrumental delivery | OR 0.96 (0.92–1.01) | 0.105 |
| Emergency cesarean section | OR 1.04 (0.99–1.08) | 0.126 |
| **Secondary outcomes** |  |  |
| Log labor duration | β 0.01 (−0.01–0.02) | 0.350 |
| Log cumulative oxytocin dose | β 0.04 (0.01–0.07) | 0.0078 |

Pre-pregnancy BMI was analyzed as a continuous variable.

Adjusted estimates were obtained from multivariable logistic regression models for binary outcomes and linear regression models for continuous outcomes.

Models were additionally adjusted for gestational weight gain (%), maternal age, parity, epidural analgesia, and infertility treatment.

For binary outcomes, results are presented as odds ratios (ORs) with 95% confidence intervals (CIs).

For continuous outcomes, results are presented as regression coefficients (β) with 95% CIs after logarithmic transformation of the outcome variables.

BMI, body mass index; GWG, gestational weight gain.

**Supplementary Table S4. Multivariable logistic regression including an interaction term between delivery BMI and birth weight for instrumental delivery**

| **Variable** | **Adjusted estimate (95% CI)** | **p value** |
| --- | --- | --- |
| Delivery BMI (per 1 kg/m²) | OR 0.96 (0.91–1.00) | 0.038 |
| Birth weight | OR 1.00 (1.00–1.001) | 0.263 |
| Delivery BMI × Birth weight | OR 1.00 (1.00–1.00) | 0.081 |

Models were adjusted for maternal age, parity, epidural analgesia, and infertility treatment. Birth weight was included as a continuous variable.

Results are presented as odds ratios (ORs) with 95% confidence intervals (CIs).

The interaction term represents effect modification between delivery BMI and birth weight.

Delivery BMI and birth weight showed a weak positive correlation (Spearman *ρ ≈ 0.24*).

BMI, body mass index.

**Table S5. Sensitivity analyses adjusting for pregnancy complications**

| **Outcome** | **Adjusted estimate (95% CI)** | **p value** |
| --- | --- | --- |
| **Primary outcomes** |  |  |
| Instrumental delivery | OR 0.96 (0.92–1.00) | 0.059 |
| Emergency cesarean section | OR 1.03 (0.99–1.07) | 0.140 |
| **Secondary outcomes** |  |  |
| Log labor duration | β 0.01 (−0.00–0.03) | 0.175 |
| Log cumulative oxytocin dose | β 0.04 (0.01–0.07) | 0.0069 |

Models were additionally adjusted for gestational diabetes mellitus, hypertensive disorders of pregnancy, fetal growth restriction, maternal medical comorbidities, and fetal anomalies, in addition to maternal age, parity, epidural analgesia, and infertility treatment.

Results are presented as odds ratios (ORs) with 95% confidence intervals (CIs) for binary outcomes and regression coefficients (β) with 95% CIs for continuous outcomes after logarithmic transformation.

Variance inflation factors for all models were <2, indicating no relevant multicollinearity.

BMI, body mass index.

**Supplementary Table S6. Sensitivity analyses restricted to inductions for post-term pregnancy, PROM, and uterine inertia (n = 607)**

| **Outcome** | **Adjusted estimate (95% CI)** | **p value** |
| --- | --- | --- |
| **Primary outcomes** |  |  |
| Instrumental delivery | OR 0.95 (0.90–0.99) | 0.026 |
| Emergency cesarean section | OR 1.05 (1.00–1.10) | 0.045 |
| **Secondary outcomes** |  |  |
| Log labor duration | β 0.02 (0.00–0.03) | 0.048 |
| Log cumulative oxytocin dose | β 0.05 (0.02–0.08) | 0.002 |

Analyses were restricted to cases induced for post-term pregnancy, premature rupture of membranes (PROM), or primary/secondary uterine inertia.

Inductions for other medical indications, including pregnancy complications and fetal indications, were excluded.

Models were adjusted for maternal age, parity, epidural analgesia, and infertility treatment.

Results are presented as odds ratios (ORs) with 95% confidence intervals (CIs) for binary outcomes and regression coefficients (β) with 95% CIs for continuous outcomes after logarithmic transformation.

BMI, body mass index.

**Supplementary Table S7. Association between delivery BMI and cumulative oxytocin dose restricted to vaginal deliveries (n = 588)**

| **Variable** | **Adjusted Estimate (95% CI)** | **p value** |
| --- | --- | --- |
| Delivery BMI (per 1 kg/m² increase) | β 0.036 (0.007–0.066) | 0.014 |

Model adjusted for maternal age, parity, epidural analgesia, and infertility treatment.
Outcome: log(cumulative oxytocin dose + 1).

**Supplementary Table S8. Stratified analyses according to pre-pregnancy BMI categories**

| **Outcome** | **preBMI <25 Adjusted estimate (95% CI)** | **preBMI <25 p value** | **preBMI ≥25 Adjusted estimate (95% CI)** | **preBMI ≥25 p value** |
| --- | --- | --- | --- | --- |
| Instrumental delivery | OR 1.00 (0.92–1.09) | 0.994 | OR 1.00 (0.91–1.10) | 0.938 |
| Emergency cesarean section | OR 1.12 (1.03–1.23) | 0.0125 | OR 1.02 (0.94–1.11) | 0.627 |
| Log labor duration | β 0.016 (-0.017–0.049) | 0.334 | β 0.018 (-0.014–0.050) | 0.279 |
| Log cumulative oxytocin dose | β -0.004 (-0.068–0.060) | 0.907 | β 0.063 (0.010–0.115) | 0.0196 |

Values are presented as odds ratios (ORs) with 95% confidence intervals (CIs) for binary outcomes and regression coefficients (β) with 95% CIs for continuous outcomes after logarithmic transformation of the outcome variables.

Pre-pregnancy BMI was categorized as <25 and ≥25 kg/m².

Stratified analyses were conducted using the same multivariable models as in the primary analysis.

Models were adjusted for maternal age, parity, epidural analgesia, and infertility treatment.

BMI, body mass index.
